# A Novel Scoring Approach for Protein Co-Purification Data Reveals High Interaction Specificity

**DOI:** 10.1371/journal.pcbi.1000515

**Published:** 2009-09-25

**Authors:** Xueping Yu, Joseph Ivanic, Anders Wallqvist, Jaques Reifman

**Affiliations:** Biotechnology HPC Software Applications Institute, Telemedicine and Advanced Technology Research Center, U.S. Army Medical Research and Materiel Command, Ft. Detrick, Maryland, United States of America; Washington University School of Medicine, United States of America

## Abstract

Large-scale protein interaction networks (PINs) have typically been discerned using affinity purification followed by mass spectrometry (AP/MS) and yeast two-hybrid (Y2H) techniques. It is generally recognized that Y2H screens detect direct binary interactions while the AP/MS method captures co-complex associations; however, the latter technique is known to yield prevalent false positives arising from a number of effects, including abundance. We describe a novel approach to compute the propensity for two proteins to co-purify in an AP/MS data set, thereby allowing us to assess the detected level of interaction specificity by analyzing the corresponding distribution of interaction scores. We find that two recent AP/MS data sets of yeast contain enrichments of specific, or high-scoring, associations as compared to commensurate random profiles, and that curated, direct physical interactions in two prominent data bases have consistently high scores. Our scored interaction data sets are generally more comprehensive than those of previous studies when compared against four diverse, high-quality reference sets. Furthermore, we find that our scored data sets are more enriched with curated, direct physical associations than Y2H sets. A high-confidence protein interaction network (PIN) derived from the AP/MS data is revealed to be highly modular, and we show that this topology is not the result of misrepresenting indirect associations as direct interactions. In fact, we propose that the modularity in Y2H data sets may be underrepresented, as they contain indirect associations that are significantly enriched with false negatives. The AP/MS PIN is also found to contain significant assortative mixing; however, in line with a previous study we confirm that Y2H interaction data show weak disassortativeness, thus revealing more clearly the distinctive natures of the interaction detection methods. We expect that our scored yeast data sets are ideal for further biological discovery and that our scoring system will prove useful for other AP/MS data sets.

## Author Summary

To understand and model cellular processes, we require accurate descriptions of the interactions occurring between constituent proteins. Large-scale protein interaction maps have typically been measured in two distinct ways. The first detects direct pair-wise associations by testing only two proteins at a time for an interaction. The second detects large groups of proteins that have conglomerated or purified together. With regard to the latter, it is difficult to deduce which pairs of proteins are physically interacting in the purification data, and interaction maps generally appear random and unstructured. We have developed a novel computational method to analyze the purification data (from the second method) and identify which proteins are directly interacting. The resultant protein interaction map is highly modular, meaning that the proteins organize themselves into localized, densely connected regions that likely represent individually functioning units. We also analyzed interaction maps of the first method and propose that their lack of modularity is a consequence of missing interactions that are undetected for unclear reasons. This study provides insights into the differences between the two interaction detection methods as well as the nature of biological organization.

## Introduction

Insights into the architectures and mechanisms of cellular processes can be obtained by elucidation of genome-wide protein interaction networks (PINs) that describe the physical associations between the component proteins. Such maps, or interactomes, can be exploited to enhance many types of biological discovery including protein function prediction [1], inference of disease genes [2], and identification of condition-specific response modules [3]. The yeast *Saccharomyces cerevisiae* has been routinely employed as a model system for high-throughput studies and PINs have been determined using a number of platforms including yeast two-hybrid (Y2H) screens [4]–[6], affinity purification followed by mass spectrometry (AP/MS) [7]–[9], and protein-fragment complementation assays (PCA) [10]. Each approach perceives interactions in a distinct manner. The Y2H and PCA techniques detect direct binary interactions, although the PCA approach does not rely upon expression of a reporter gene as required in Y2H screens, while the AP/MS techniques purify and identify protein complexes. The reliability of each technique has been extensively debated in the literature and comprehensive analyses have resulted in contrasting conclusions [6], [10]–[12]. However, it is generally accepted that any measure of reliability is not absolute and largely dependent on the nature of a pre-defined gold standard reference set.

An additional complexity arises in the analysis, or interpretation, of an AP/MS data set because there is no standard, or well-defined, system to distinguish between the direct and indirect interactions present in a purified complex. The only information available for an individual purification is its composition: a tagged bait protein and associated co-purified prey proteins. Furthermore, the constituent proteins are identified by complex MS methods and different platforms often yield varying compositions for identical purifications [9],[13]. Another concern is that the compositions of the purifications are influenced by the protein abundances [11],[14],[15] - proteins having a higher abundance are more likely to be detected in more purifications and, therefore, inferred to be involved in more interactions after tabulation of all bait-prey pairs [15]. To address these issues, a number of approaches for the analysis of AP/MS data sets have been employed [8],[9], [16], [17]. These techniques have the common goal of discerning protein pairs that are appreciably co-purified relative to some random background. While each method determines scores representing the likelihood of observing two proteins together, the scores are computed using different procedures: Gavin et al. calculate log-ratios of observed co-occurrences relative to expected [8]; Krogan et al. utilize a combination of machine learning algorithms [9]; Collins et al. implement a supervised algorithm derived from Bayesian methods and optimized with empirically-derived parameters [16]; and Hart et al. determine interaction probabilities based on hypergeometric distributions [17]. The qualities of the generated PINs have been found to be superior to comparable data sets constructed by straightforward tabulations of bait-prey interactions [9],[16],[17]. These evaluations were generally deduced from direct comparisons against complexes manually curated by the Munich Information Center on Protein Sequences (MIPS) [18].

A recent study of high-throughput Y2H data sets explored the characteristic strengths and distributions of functional (specific) interactions and non-functional (non-specific or transient) interactions in order to assess the extent to which the latter impedes the formation of functional protein complexes [19]. It was conjectured that the overall impact upon biochemical efficiencies had evolved to a tolerable limit.

Motivated by the use of randomization techniques as a tool to measure, or discover, enrichments of network motifs [20] and connectivity correlations [21] in complex networks, we developed a shuffling-based approach to assess the levels of interaction specificity detected in AP/MS data sets. This system allows for the computation of pair-wise protein co-occurrence significance (CS) scores by comparing experimentally observed numbers with those from randomized realizations. A CS score for two proteins provides a statistical measure of their propensity to co-purify, or interact, in an AP/MS data set. The approach requires no training set or machine learning and is, therefore, applicable to any AP/MS data set for any species regardless of whether any curated information exists or not. It is found that these AP/MS data sets contain significant enrichments of specific, or high-scoring, associations. Additionally, we showed that high-quality direct physical interactions curated in two prominent data bases have significantly high CS scores. Therefore, while the AP/MS data sets contain prevalent non-specific, or transient, associations, our scoring analysis reveals that there is an underlying preference for proteins to form selective, or discriminating, associations. Our resultant scored interaction data sets were further assessed by comparisons against four diverse, high-quality reference data sets, each representing a unique manner of interaction detection, association mechanism (direct or co-complex), and/or curation. For most references, we found that the accuracies of our scored interaction sets were manifestly higher than those of previous studies. Additionally, our scored data sets are the only ones that typically outperformed experimental Y2H interaction sets [4]–[6]. A high-confidence PIN extracted from the AP/MS data of Gavin et al. [8] was revealed to be free of abundance effects while those derived from the data of Krogan et al. [9] contained weak abundance biases. Therefore, it would appear that in high-quality AP/MS data sets, interaction specificity is not coupled with protein abundance. We note that the converse has recently been found to be true of Y2H interaction data sets [19].

The high-confidence PIN derived from the data of Gavin et al. [8] was shown to be highly modular, containing many localized densely-connected regions, and strikingly different to a commensurate random network. We also demonstrated that the observed high modularity is not a result of misinterpreting indirect associations as direct interactions; rather, it is a result of direct physical associations. Furthermore, we suggest that the modularity in Y2H interaction data sets may be underrepresented as indirect associations in these PINs are significantly enriched with manually-curated physical interactions, i.e., they are likely false negatives.

The high-confidence AP/MS PIN shows assortative mixing, meaning that proteins having similar numbers of total interactions prefer to interact with each other. A consequence of assortativity is that high-degree proteins, or hubs, prefer to associate with each other rather than with proteins having very small numbers of total interactions. In agreement with a previous study [21], we find that a consolidated Y2H PIN shows weak disassortative mixing while a manually-curated set of high-confidence physical binary interactions displays both, and in equal measure, assortative and disassortative mixing. Therefore, high-quality AP/MS data appear assortative while Y2H interaction data appear disassortative.

We expect that our scored yeast data sets are ideal for further investigations involving biological discovery and that our procedure will prove useful for the analysis of current and future AP/MS data sets for a variety of species. We have compared our high-quality AP/MS interaction data sets with those from Y2H screens and perceived a number of novel insights regarding their substances and network properties. Certainly, their topologies are contrasting and must reflect their different methods of interaction detection.

## Materials and Methods

### Calculation of Co-Occurrence Significance Scores

A CS score is a measure of the propensity for two proteins to be identified together in purifications, either as bait-prey or prey-prey combinations, relative to what would be expected by chance. They were determined by comparing observed co-occurrences, the number of times two proteins coincided in purifications, with those from random simulations, where the latter were realized by thoroughly shuffling, or exchanging, prey proteins (see below). Therefore, our CS scores are derived from a purely numerical procedure and, unlike previous systems of Krogan et al. [9] and Collins et al. [16], require no training or reference data sets. Our CS scores are related to the socio-affinity indices of Gavin et al. [8] and the probabilistic scoring scheme of Hart et al. [17] in that they attempt to quantify the propensity for proteins to co-purify. The socio-affinity scoring system [8] uses log-ratios of actual co-occurrences relative to what would be expected based upon protein purification frequencies, while the probabilistic scoring scheme [17] calculates interaction scores based upon hypergeometric distributions. However, both of these methods use expected occurrence baselines determined from total numbers of protein populations or interactions. As such, they do not account for the great variations in bait affinities, i.e., the observation that some bait proteins purify with very many preys while others purify with very few.

Our procedure is distinct in that we determine numbers of expected, or chance, co-occurrences via constrained randomized simulations that preserve the individual purification structures, i.e., the number of preys. Although simplistic in its nature, our scoring system is advantageous in several ways. First, the method generates co-occurrence distributions for each protein pair and, therefore, is able to gauge the statistical significances of the actual experimentally observed co-occurrences. Second, while the method penalizes proteins having higher frequencies of purification, or abundances, it is able to uniformly distinguish between specific and indiscriminate partnerships. In fact, the method is able to identify instances of negative associations, or protein pairs that have significantly under-represented observed co-occurrences relative to that expected. Third, our randomized simulations preserve the numbers of proteins in the individual purifications and, consequently, utilize the experimentally discerned affinities of the bait proteins. Last, as mentioned above, the procedure is purely numerical and does not require a training or reference data set. Therefore, it is completely devoid of any associated bias and is applicable to any affinity purification data set, regardless of whether any other high-confidence interaction sets exist or not.

**Figure 1:**
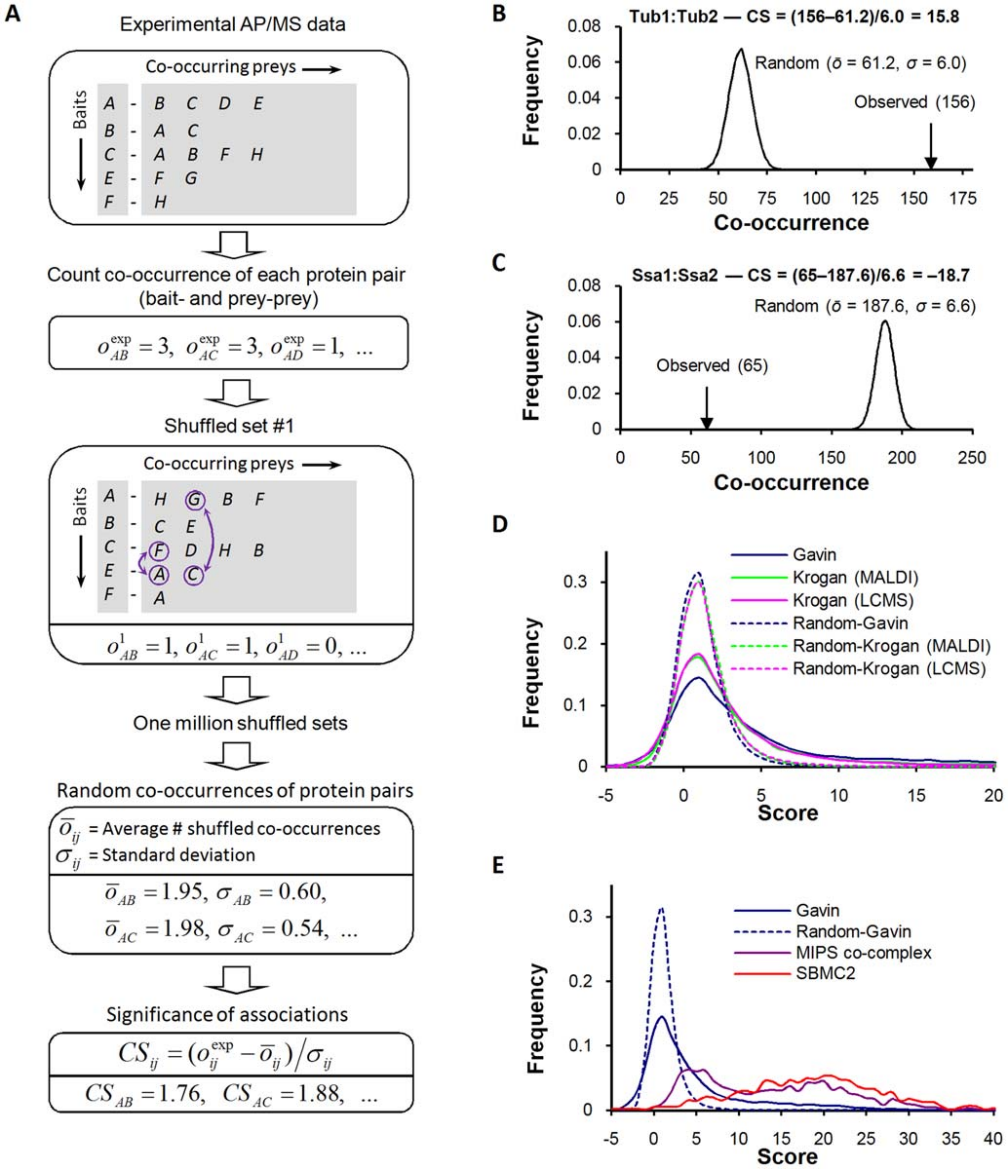
Co-occurrence significance (CS) scores measure the interaction specificity for two proteins in AP/MS data. (A) Flow chart for the computation of CS scores. (B) Illustration for protein pair Tub1∶Tub2 showing an overrepresented co-occurrence in the purification data of Gavin et al. [8]: 156 (observed) vs. 61.2 (*σ* = 6.0) (random), with corresponding CS score of 15.8. (C) Illustration for protein pair Ssa1∶Ssa2, showing an underrepresented co-occurrence in the purification data of Gavin et al. [8]: 65 (observed) vs. 187.6 (*σ* = 6.6) (random), with corresponding CS score of −18.7. (D) Total score distributions of experimental data sets and corresponding average distributions from 10^5^ random (shuffled) realizations (see Materials and Methods). (E) score distributions, in purification data of Gavin et al. [8], of selected curated interactions in MIPS [18] and SGD-Biogrid (SBMC2) [23],[24] repositories (see Materials and Methods) showing their measured high specificities.

Our interaction detection based on shuffling (IDBOS) procedure is depicted in Figure 1A. For a given affinity purification data set in which individual purifications are specified by a bait protein and co-purifying prey proteins, we counted, for each unique protein pair *i* and *j*, the total number of times they co-occurred in the same purification. These observed co-occurrences, *o_ij_*, do not distinguish between bait-prey or prey-prey combinations. We then constructed randomized, or shuffled, purification sets and computed average shuffled co-occurrences, *ō_ij_*, and associated standard deviations, *σ_ij_*. The CS score for each protein pair was then determined as the Z-score of the observed co-occurrences:
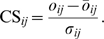
(1)A shuffled purification set was constructed by shuffling, or exchanging, pairs of prey proteins in a reference data set. A single realization was accomplished by enumerating all prey proteins (in all purifications) once and, for each prey protein, exchanging it with another prey protein chosen at random. However, an exchange was subject to the following constraints: (i) the two prey proteins must occur in different purifications, and (ii) the exchange cannot result in any purification having a protein that appears twice, whether as bait or prey. This construction procedure ensured that the shuffled purification sets were comparable to the experimental data set, whereby the numbers of proteins in the individual purifications were conserved and the global population of each protein remained unchanged. We constructed a million shuffled sets for each affinity purification data set analyzed here. An initial shuffled set was derived directly from the experimental purification data and subsequent shuffled sets were derived from ones immediately previous.

When tabulating CS scores of protein pairs, or interactions, derived from an experimental affinity purification data set, we retained only those for observed co-occurrences greater than one, i.e., *o_ij_*>1. We deemed that statistical significances of protein associations having co-occurrences less than two were not as reliable as those having higher co-occurrences. However, we stored mean shuffled co-occurrences and associated standard deviations computed from the million shuffled sets for all possible protein pairs. These were used to gauge the distribution of the tabulated CS scores through the following steps. First, an additional 10^5^ shuffled sets were constructed in the same manner as that described above. Second, for each shuffled set, we determined the Z-scores for protein pairs having a shuffled co-occurrence of greater than one:
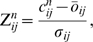
(2)where 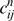 (>1) is the co-occurrence of proteins *i* and *j* in the *n*th shuffled set, and *ō_ij_* and *σ_ij_* are the mean co-occurrences and standard deviations, respectively, determined from the million shuffled sets as in Equation (1). The total shuffled distribution, comprising Z-scores accumulated from the 10^5^ shuffled sets, was used as a baseline to contrast the distribution of CS scores.

### Evaluation of Interaction Data Sets

A standard way to evaluate an interaction data set is to contrast it against a reference set that is considered to be high quality. Commensurate with a previous approach [22], we have computed accuracy versus coverage, where coverage is the number of coinciding interactions in the evaluated and reference sets and accuracy is the fraction of interactions in the evaluated set that are coincident. When an interaction data set included confidence scores, as in the sets derived in this work and in previous studies [8],[9],[16],[17], we ranked the interactions by decreasing score and plotted accuracy versus coverage curves over a range of score cutoffs.

We used four reference interaction data sets that are each considered to be high quality in some way. However, they are also individually distinct in that each represents a different style of interaction measurement or curation. By evaluating, or contrasting, interaction data sets against these references, we were able to assess their substances from a number of viewpoints. Descriptions of the reference sets follow:

- The binary gold standard (BGS) data set is a manually curated set of high-confidence physical binary interactions that represent direct protein associations, rather than indirect ones, that may be incorporated in co-complex AP/MS data sets [6]. This interaction set has been shown to have considerable overlaps with high-throughput Y2H data sets.
- A recent PCA strategy detects *in vivo* protein interactions via fusions to enzyme fragments that, when reconstituted, restores catalytic activity and, consequently, cell growth. Therefore, this PCA approach does not depend upon the expression of a reporter protein as required in Y2H screens [10]. This PCA technique was applied on a genome-wide scale for yeast and yielded many new, previously undiscovered protein interactions.
- The Saccharomyces Genome Database [23], (SGD: http://downloads.yeastgenome.org/literature_curation/interaction_data.tab), which coincides with binary interactions in the general repository of interaction data (BioGRID) [24], was mined for ‘physical interactions’ that were ‘manually-curated’ and reported at least twice. We removed from this subset the PCA interaction data described immediately above. The resulting data set is referred to as SBMC2.
- MIPS curated complexes were downloaded from the MIPS database (ftp://ftpmips.gsf.de/yeast/catalogues/complexcat) [18]. We only considered complexes identified in low-throughput experiments, i.e., complexes listed under category 550, labeled as ‘Complexes by Systematic Analysis,’ were excluded. Only pairs of proteins belonging to the same complex were considered as interacting.

### Y2H Interaction Data Sets

We also analyzed yeast PINs determined from a number of high-throughput Y2H screens in order to contrast their contents and network structures against the scored AP/MS data sets. The Y2H data sets studied included the interaction sets of Yu et al. [6] (CCSB-YI1), Ito et al. [4] (core subset), Uetz et al. [5], and a union of these sets [6] (Y2H-union).

### Network Analysis

The network structures of protein interaction data sets were analyzed by computing a variety of graph-theoretical properties. The clustering coefficient of a node (or protein) *i* is defined as the fraction of possible edges between neighbors that are present, where a neighbor of node *i* is any other node that shares an edge with it [25]. The average clustering coefficient of a network was determined by averaging the clustering coefficients of all nodes, where nodes involved in only one interaction are defined here to have a clustering coefficient of zero. The clustering coefficient of a network is an indication of the network's modularity, although it is not a strict measure.

The nature of the connectivity in a network was assessed here by determining interaction frequencies between pairs of degrees, i.e., for two degrees *k*_1_ and *k*_2_, we counted the total number of interactions occurring between two nodes where one has degree *k*_1_ and the other has degree *k*_2_. Enrichments of interaction frequencies between degrees were measured as Z-scores, where actual numbers were compared to those of commensurate, randomly-rewired, degree-preserving networks (10^3^ realizations) that were constructed using a similar procedure to that of Maslov and Sneppen [21]. To verify our interpretation of the interaction frequencies with regards to the connectivity in a network, we also computed the degree-degree correlation coefficient [26],[27], which quantifies the level of interaction between proteins of similar degrees:
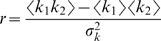
(3)where the averaged quantities are determined over all interactions and the denominator is the variance of the node degree *k*. When nodes of similar degrees prefer to interact in a network, i.e., their interaction frequencies are significantly enriched resulting in a positive degree-degree correlation coefficient (*r*>0), then the network connectivity is said to be assortative – nodes of high degree (hubs) prefer to interact with each other while low-degree nodes avoid interacting with hubs. Conversely, when nodes of diverse degrees prefer to interact in a network, leading to a negative correlation coefficient (*r*<0), then the connectivity is said to be disassortative – hubs avoid each other and generally prefer to interact with low-degree proteins.

## Results

### Computation and Analysis of Co-Occurrence Significance Scores

We applied our IDBOS scoring procedure (Figure 1A and see Materials and Methods) to the yeast AP/MS experimental data sets of Gavin et al. [8] and Krogan et al. [9]. Gavin et al. used matrix-assisted laser desorption/ionization-time of flight (MALDI-TOF) MS to identify proteins present in the purification while Krogan et al. used two MS techniques for protein identifications: MALDI-TOF and liquid chromatography tandem MS (LCMS). Although previous studies have merged the MALDI-TOF and LCMS data sets of Krogan et al., we chose to keep them separate initially. Therefore, we computed three sets of CS scores for each of the Gavin, Krogan (MALDI-TOF), and Krogan (LCMS) AP/MS data sets that formed our IDBOS-Gavin, IDBOS-Krogan (MALDI), and IDBOS-Krogan (LCMS) scored interaction data sets, respectively (Tables S1, S2, S3). Only CS scores for protein pairs having total co-occurrences greater than one were retained. As discussed above (see Materials and Methods), the CS score for a protein pair represents the propensity for them to co-purify (or associate) relative to a random background derived from simulations that shuffled prey proteins.

We illustrate the approach for the two proteins Tub1 (YML085C) and Tub2 (YFL037W) that are known to form alpha and beta subunits of heterodimers that polymerize to form microtubules. These cytoskeletal filaments participate in a variety of cellular functions, including structural support [28]. The significance of the Tub1–Tub2 associations in the AP/MS data set of Gavin et al., i.e., the CS score in the IDBOS-Gavin data set, is shown in Figure 1B. The random profile has a mean co-occurrence of 61.2 and a standard deviation of 6.0, indicating that the observed co-occurrence, or frequency of co-purification, at 156 is statistically significant with a Z-score of 15.8. Therefore, we would consider that Tub1 and Tub2 have a high affinity of association. In contrast, previous analyses of the Gavin et al. data set have not concluded that these two proteins have a significant association [8],[16]. In fact, only the study of Hart et al. [17] infers a significant association for these two proteins; however, some of their scores are computed by multiplying *P*-values across data sets. Curiously, the interaction between Tub1 and Tub2 has not been identified in any of the high-throughput Y2H or PCA screens [4]–[6],[10].

Perhaps a more intriguing illustration of our approach is the discerned highly-specific non-interaction, or perceived repulsive association, between the two proteins Ssa1 (YAL005C) and Ssa2 (YLL024C). These proteins have an experimentally observed co-occurrence of 65 in the AP/MS data set of Gavin et al. while the random profile has a mean-co-occurrence of 187.6 and a standard deviation of 6.6; therefore, the resultant CS score is considerably negative at −18.7 (Figure 1C). This score implies that not only do these proteins not interact; they would rather not associate, even by chance. The reasons for this inferred repulsive association are not immediately clear. Ssa1 and Ssa2 are cytosolic members of the heat shock protein 70 family that have a number of functions, including serving as molecular chaperones and assisting in protein folding [29]. A possible explanation for their avoidance may be to enhance their protein translocation efficiencies – if they were to come together, even by chance, their individual abilities to function as chaperones may be lost. It is also possible that Ssa1 and Ssa2 interact with diverse sets of proteins, i.e., Ssa1 may interact strongly with a particular set of proteins whereas Ssa2 may interact with a different group. While there has been much focus recently on elucidating the high-confidence or steadfast interactions in experimental interaction data sets, little effort has been made to identify proteins that strongly avoid each other. It remains to be seen whether this latter type of non-interaction amongst proteins is also fundamental for normal cellular function.

Although the random co-occurrence profiles for the Tub1–Tub2 and Ssa1–Ssa2 cases discussed above appear to be normally distributed (Figures 1B and 1C), it should be noted that as the average random co-occurrence for two proteins approaches zero, the corresponding random co-occurrence profile will become less normal and skewed to the right. Therefore, one may query the reliability, or appropriateness, of CS scores in such instances. We have somewhat diminished this concern by only scoring protein associations that have an observed co-occurrence of two or more (see Materials and Methods). However, we recognize that in some instances random co-occurrence profiles will deviate from normality. Nonetheless, as a starting point for more advanced (and possibly computationally inefficient) approaches, we analyzed the performance of the current procedure.

The number of potential protein pairs in an AP/MS data set is very large, in the millions for the three analyzed in this work. As such, it is possible for pairs to have significant scores for their co-occurrences purely by chance. To investigate this likelihood we contrasted the distributions of the CS scores in the three IDBOS data sets against shuffled, or random, score distributions accumulated from 10^5^ commensurate shuffled sets (see Materials and Methods). We found that the experimental distributions have longer tails in the high-score region (Figure 1D), indicating that they are enriched with discriminating protein associations. These results are encouraging in that they reveal, in a unique way, perceptible levels of specificity in the associations detected by the AP/MS experiments. Furthermore, all three random distributions are nearly identical, indicating that we are using consistent random baselines in our approach. We note that of the three experimental distributions, the IDBOS-Gavin data set has the most pronounced enrichment in the high-score region, possibly suggesting differences in the qualities of the experimental data. This issue is discussed in more detail later.

Careful examinations of the randomized Z-score distributions indicate that they deviate slightly from normality in that they are slightly skewed to the right. This is most likely a result of only scoring interactions that have a co-occurrence of two or greater in any of the experimental or the additional 10^5^ randomized data sets, i.e., for a given data set, whether experimental or one of the additional randomized, Z-scores were only determined for protein pairs that had co-occurrences of two or greater in that data set (see Materials and Methods). Therefore, the experimental and random score distributions are slightly skewed to the right. Even so, when contrasted against the random score distributions, the experimental distributions are noticeably enriched in the high-score region.

As a first step to analyzing the reliability of our scoring scheme, we gauged the scored interactions in the IDBOS-Gavin data set by mapping them on to curated interactions that represent high-confidence associations identified in small-scale, or low-throughput, experiments. For a given curated data set, we tabulated their IDBOS-Gavin scores, i.e., we accumulated IDBOS-Gavin scores for interactions that occurred in both the curated data set and our IDBOS-Gavin scored set. If the curated set contains steadfast interactions *and* our procedure is able to identify them as being statistically over-represented in the AP/MS data set of Gavin et al., then the accumulated score distribution should reflect this. Indeed, we discovered that interactions in two prominent curated sets have distinctively high CS scores in the data set of Gavin et al. (Figure 1E). The first curated set is a collection of interactions between proteins occurring in the same MIPS annotated complex (see Materials and Methods) and this data shows two peaks near CS scores of five and twenty. The distribution about five may be due to the nature of the interaction tabulation. We inferred that all proteins occurring in the same MIPS complex interact; however, most likely many of these pairs do not have a direct physical association. The second curated set is a collection of manually-curated physical interactions reported twice or more in the SGD-BioGRID repositories (see Materials and Methods). This set of interactions (SMBC2) has a CS score distribution that is also well separated from the total experimental and shuffled distributions and, like the MIPS data, exhibits a peak near twenty. Therefore, we concluded that our IDBOS scoring scheme was able to reliably distinguish between the specific and non-specific associations detected in the AP/MS experiments.

### Evaluation of the IDBOS Scoring Procedure

**Figure 2:**
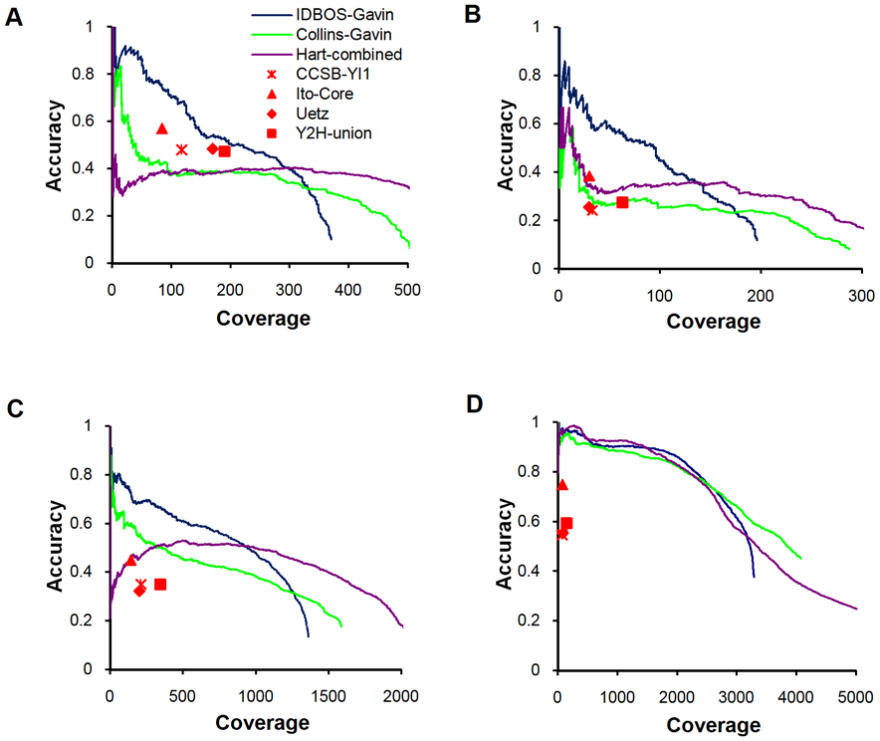
Evaluation of the IDBOS scoring scheme. Coverage versus accuracy data (see Materials and Methods) comparing the scoring schemes of IDBOS (this work) and Collins et al. [16], when applied to the purification data of Gavin et al. [8]. Four diverse reference interaction data sets were used: (A) BGS; (B) PCA; (C) SBMC2; and (D) MIPS. See Materials and Methods for full descriptions of these references. Also shown is the scored data of Hart et al. [17] (determined by multiplying individual results across the Gavin et al. [8], Krogan et al. [9], and Ho et al. [7] AP/MS data sets) and evaluations for Y2H data sets of Yu et al. [6] (CCSB-YI1), Ito et al. [4] (core subset), Uetz et al. [5], and a union of these data sets [6] (Y2H-union).

To further evaluate the IDBOS procedure we compared its performance against the previously described scoring systems of Collins et al. [16] and Hart et al. [17] by contrasting each against a variety of reference interaction sets. Both systems of Collins et al. and Hart et al. have been shown [16],[17] to out-perform the high-confidence PINs derived in the original AP/MS studies [8],[9]. Collins et al. provide purification enrichment (PE) scores computed independently for the AP/MS data sets of Gavin et al. [8] and Krogan et al. [9]; however, they analyze the latter by combining the original MALDI-TOF and LCMS purifications into one data set. Hart et al. [17] only provide scores determined by multiplying individual results across the Gavin et al. [8], Krogan et al. [9], and Ho et al. [7] data sets. Since consolidated data sets generally show greater accuracy than individual ones [16],[17], we felt that comparison of IDBOS-Gavin and Collins-Gavin interaction data against the combined data of Hart et al. [17] advantaged the latter. Accuracy versus coverage curves using four diverse reference sets are shown in Figures 2A–D (see Materials and Methods for fuller descriptions of the references and evaluation procedure). The first two references represent high-quality direct physical interactions that were either curated binary gold standard (BGS) [6] (Figure 2A) or detected in a large-scale experiment (PCA) [10] (Figure 2B). In each instance, we found that IDBOS-Gavin scored data performed better than the Collins-Gavin and Hart data sets. Similar results were obtained for the third reference (Figure 2C), which consists of manually-curated physical interactions detected in small-scale experiments (SBMC2) [23],[24]. These results suggest that our method was more adept at discerning the direct associations from the indirect that are present in the purifications. The fourth reference is a collection of interactions between proteins co-occurring in MIPS curated complexes identified in low-throughput experiments. All three scoring schemes show very high overlaps (Figure 2D) and this is probably not unexpected. By assuming that all proteins comprising a complex are interacting, we are not distinguishing between the direct and indirect associations. However, the results are encouraging for the IDBOS and Hart et al. [17] approaches as neither relies upon external data, while the method of Collins et al. [16] employed empirical parameters that were optimized using MIPS complexes. Very similar results were observed when analyzing the IDBOS- and Collins-scored data of Krogan et al. (Figure S1).

While our technique for the analysis of AP/MS data sets compares favorably with previous methods, it is of interest to contrast our scored interaction data sets against those from high-throughput Y2H screens [4]–[6]. It has recently been surmised that AP/MS methods are best at detecting co-complex associations while Y2H screens are better at detecting binary interactions when compared against the BGS set [6]. When using this BGS set as a reference, we found that the Y2H interaction sets show better relative accuracies than the Collins-Gavin and Hart data sets. However, our IDBOS-Gavin data set performed at a slightly higher level than the Y2H interaction sets (Figure 2A), although the differences are small. Nonetheless, the result further affirmed that the IDBOS procedure discerned direct physical associations in the AP/MS purification data. The IDBOS-Gavin set performed markedly better than the Y2H data sets for the other references (Figures 2B–D). The results are not unexpected when using the MIPS reference, but noteworthy for the others as they represent distinct types of high-quality direct binary interactions. Although the IDBOS-Krogan data is of slightly poorer quality than the IDBOS-Gavin data, the comparisons against the Y2H interaction sets yielded comparable results (Figure S1).

### High-Confidence AP/MS PINs

We determined score cutoffs for each IDBOS data set by comparisons of their experimental and random score distributions (see Materials and Methods) shown in Figure 1D. For a given score threshold *ζ*, we can compute the fractions of protein pairs in the commensurate random and experimental distributions that have a higher score as *f*_R_(Z>*ζ*) and *f*_E_(CS>*ζ*), respectively. Therefore, we approximated the false-discovery rate as the ratio of these fractions, i.e., *P*_FP_(*ζ*) = *f*_R_/*f*_E_. We used a false-discovery rate of 5% to compute score cutoffs for the IDBOS-Gavin (*ζ*_0.05_ = 5.95), IDBOS-Krogan (MALDI) (*ζ*_0.05_ = 8.26), and IDBOS-Krogan (LCMS) (*ζ*_0.05_ = 12.92) data sets. Corresponding high-confidence PINs were compiled by including only interactions having higher CS scores than the respective cutoffs. The number of proteins/interactions in the IDBOS-Gavin, IDBOS-Krogan (MALDI), and IDBOS-Krogan (LCMS) PINs were 1274/7879, 1061/3398, and 1719/3640, respectively. The IDBOS-Gavin PIN has the largest number of interactions of the three, which demonstrated the superior enrichment of high CS scores in the AP/MS data set of Gavin et al. [8]. The IDBOS-Krogan (LCMS) PIN is the sparsest, as judged by the average number of interactions, or degree, of the constituent proteins, implying that the LCMS data of Krogan et al. [9] has the lowest enrichment of significant association scores. Certainly, these observations are mirrored by the order of the computed score cutoffs given above.

**Figure 3:**
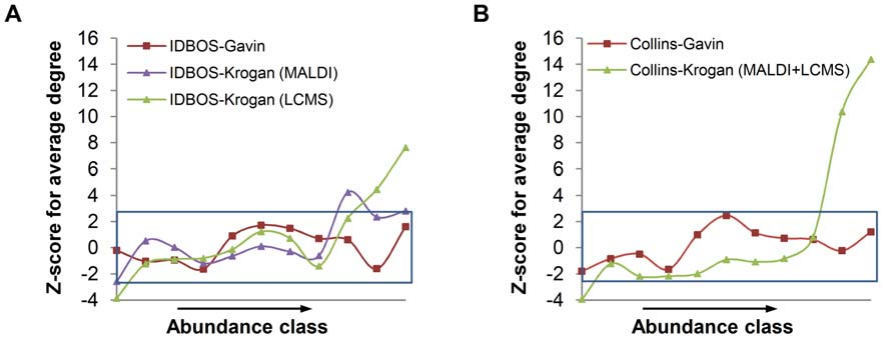
Abundance effects in high-confidence PINs derived from AP/MS data. The association between protein degree and abundance in high-confidence PINs derived by (A) the IDBOS procedure (this work) and (B) Collins et al. [16], from AP/MS data sets of Gavin et al. [8] and Krogan et al. [9]. Proteins were sorted by increasing abundance, as measured by Newman et al. [30], into 11 classes. Undetectable low-abundant proteins comprised class 0 while the remaining proteins were sorted into 10 equally-sized classes. The sizes of classes 0/classes 1–10 were as follows: 231/92 for the IDBOS-Gavin PIN; 265/68 for the IDBOS-Krogan (MALDI) PIN; 424/101 for the IDBOS-Krogan (LCMS) PIN; 238/87 for the Collins-Gavin PIN; and 384/111 for the Collins-Krogan (MALDI+LCMS) PIN. For each class, we determined the significance of the average degree, as a Z-score, compared to the network average and standard deviation determined from equivalently-sized randomly-compiled pools (10^4^ realizations). The enclosed rectangular areas represent |Z|<2.6 (P>0.05 after multiple-test correction).

From the results presented so far, one might conclude that of the three AP/MS data sets investigated here, the set of Gavin et al. [8] showed the highest specificity of protein associations: (i) it had the most considerable enrichment of high CS scores (Figure 1D) and, consequently, it yielded the most interactions from use of a 5% false-discovery-rate filter; and (ii) the IDBOS-Gavin and Collins-Gavin scored data sets generally showed superior performance over the comparable scored sets derived from the data of Krogan et al. [9] (Figure 2 and Figure S1). We investigated this premise further by analysis of protein abundance trends in the high-confidence PINs derived in this work and by Collins et al. [16]. It has previously been demonstrated that proteins having higher cellular abundances tend to be involved in more interactions, or have higher degrees, in AP/MS experimental data sets; such an abundance-degree relationship is not present in PINs determined from Y2H screens [11],[14],[15]. Abundance effects were assessed using an approach similar to that of von Merring et al. [11], whereby proteins in a PIN were sorted into classes according to their abundances. We utilized the recent abundance measurements of Newman et al. determined from flow cytometry [30]; however, similar results were observed when using abundances measured by western blot analysis [31] (data not shown). We found that the IDBOS-Gavin PIN is free of any abundance effects while the IDBOS-Krogan (LCMS) PIN shows a weak bias in the high-abundance/high-degree region (Figure 3A). Equivalent results were obtained when we analyzed the high-confidence networks of Collins et al. [16] (Figure 3B), which were each constructed using the score cutoff of 3.19 used for their merged data. Like our IDBOS-Gavin PIN, the high-confidence Collins-Gavin network shows no significant abundance effects. We could only construct a merged Collins-Krogan (MALDI+LCMS) PIN from their available data and this network shows the largest high-abundance/high-degree bias.

The observation that only the high-confidence PINs derived from the results of Gavin et al. [8] are free of any abundance bias is consequential. This finding, together with those discussed earlier, imply that the AP/MS experiment of Gavin et al. [8] detected more specific protein associations than that of Krogan et al. [9]. For the latter study, the score-enrichment and abundance analyses described above indicate that the MALDI-TOF method identified more specific associations than the LCMS technique. However, we do not wish to make firm conclusions regarding the two identification methods. There are other important factors that we have not considered, not least that the LCMS method is purported to be more successful in identifying small and lower-abundance proteins [9],[13]. Such an advantage might certainly lead to a perceived lower-specificity, at least by the analysis methods used here, simply because more unique proteins may be detected.

### Architecture of High-Confidence AP/MS PINs

**Figure 4:**
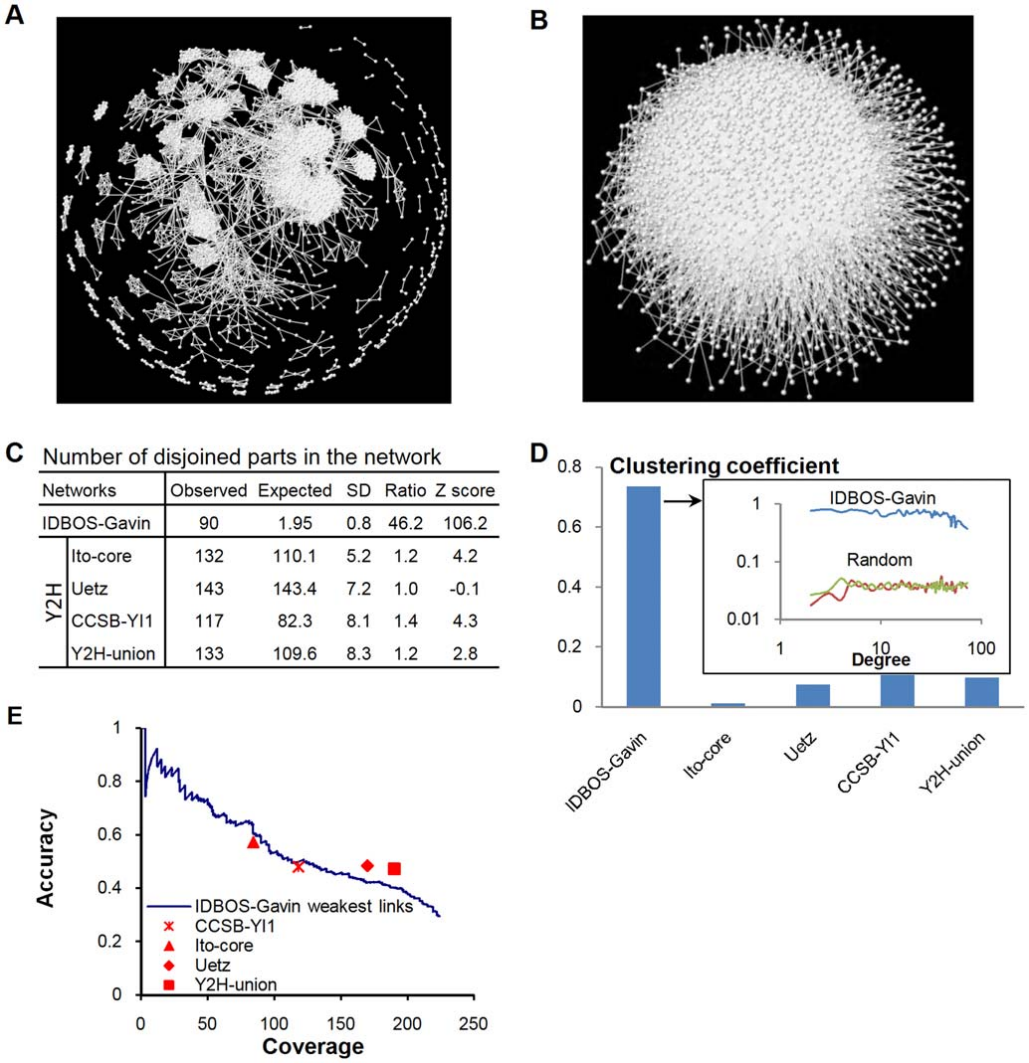
The high-confidence IDBOS-Gavin PIN is highly modular. Depictions of (A) the high-confidence IDBOS-Gavin PIN and (B) a commensurate, degree-preserving random network. (C) Enrichments of numbers of disjoined parts in the IDBOS-Gavin PIN and Y2H data sets of Yu et al. [6] (CCSB-YI1), Ito et al. [4] (core subset), Uetz et al. [5], and a union of these data sets [6] (Y2H-union). Expected values and standard deviations (SD) were computed from 1000 realizations of commensurate, degree-preserving random networks. (D) Clustering coefficients of the IDBOS-Gavin PIN and experimental Y2H data sets. The inset shows average clustering coefficients by degree for the IDBOS-Gavin PIN and two realizations of a commensurate, degree-preserving random network. (E) Coverage versus accuracy data for the weakest links in the IDBOS-Gavin PIN using the BGS reference set (see Materials and Methods). Also shown are coverage-accuracy values for the Y2H data sets.

Although the primary focus of the present article is the description and analysis of the IDBOS scoring procedure for AP/MS data, it is useful to examine the network structures of the derived high-confidence PINs. Since our evaluations suggest, but certainly not affirm, that the AP/MS data of Gavin et al. [8] contains more specific protein associations than the data sets of Krogan et al. [9], we opt to present network analyses of the high-confidence IDBOS-Gavin PIN described above; however, the IDBOS-Krogan PINs show very similar characteristics. The IDBOS-Gavin PIN is depicted in Figure 4A and its modular nature is immediately apparent. We want to make it clear that in this work we have strictly not quantified the levels of modularity in any network. Rather, we have inferred modular natures, or lack of, via a number of graph-theoretical analyses and illustrations. While a refined two-dimensional portrayal of a network can reveal the inherent modularity, it often also disperses modules that are incorporated in the giant component. Nonetheless, it is clear from Figure 4A that the IDBOS-Gavin PIN contains many localized highly-clustered regions as well as numerous disjoined complexes. The IDBOS-Gavin PIN is strikingly different to a commensurate randomly-rewired, degree-preserving network (constructed using a similar procedure to that of Maslov and Sneppen [21]), which shows no modularity or disjoined regions (Figure 4B). Interaction data sets generated from the raw AP/MS data of Gavin et al. [8], using the spoke (bait-prey tabulation) and matrix (bait-prey and prey-prey tabulation) models, appear very similar to the random in that they exhibit very little modularity and appear uniformly dense (Figure S2). While the number of disjoined components in a network, relative to that of a commensurate random network, is not a strict measure of modularity, it does provide insight into the level of interaction localization. The IDBOS-Gavin PIN has 90 disjoined components compared to the expected 1.95 (SD = 0.8) for the random equivalent based on 1000 realizations (Figure 4C). This substantial 46-fold increase clearly indicates preferential protein complexation. In contrast, the Y2H interaction networks show no significant enrichments of disjoined components with observed/expected ratios of close to one (Figure 4C). Therefore, the IDBOS-Gavin PIN shows a much higher level of selective complexation than the Y2H data sets. While this is to be expected due to the nature of the AP/MS method, the results imply that the IDBOS scoring procedure was able to identify individual complexes occurring in the purification data. Another indicator of the level of modularity in a network is the average clustering coefficient of a network which is literally a measure of edge clustering around the nodes or proteins [25]. We averaged clustering coefficients over proteins in a PIN having degrees greater than one. The IDBOS-Gavin PIN has an average clustering coefficient of 0.74 and this is much higher than those for the Y2H interaction data sets (Figure 4D). This 19-fold ratio of observed relative to commensurate random suggests a significant enrichment of clustering. Of the Y2H interaction sets, the Uetz et al. [5] data set has the highest ratio of observed/expected of 14 while the core PIN of Ito et al. [4] has the lowest of approximately one. Therefore, the Y2H PIN of Uetz et al. [5] also shows a significant clustering enrichment. Figure 4D shows the average clustering coefficients of proteins by degree for the IDBOS-Gavin PIN and two realizations of a commensurate random network. It is clear that the clustering tendency of a protein in the IDBOS-Gavin PIN is essentially independent of its degree, only dropping slightly at very high degrees, and is substantially higher than the random. The clustering profile in the IDBOS-Gavin PIN is manifestly different from the power-law profile of hierarchical networks previously proposed to model biological networks having power-law-like degree distributions [32],[33]. Although the IDBOS-Gavin PIN is also characterized by a power-law-like degree distribution (see *High-Confidence AP/MS PINs Show Assortative Mixing*), it is clear that this network does not have a hierarchical structure (Figures 4A and D) and that the IDBOS scoring procedure is discerning an inherent modular nature for the preferential protein interactions in the AP/MS purifications.

### Direct Versus Indirect Associations in AP/MS and Y2H PINs

It has previously been concluded that, generally, Y2H interaction sets consist of high-quality direct binary associations while AP/MS data sets contain complexes composed of direct and preponderant indirect associations [6]. Therefore, it is possible that our scoring system assigns artificially high scores to pairs of proteins occurring in the same complex, but that are not directly physically interacting. We assessed the scope of these misrepresented indirect associations in our high-confidence IDBOS-Gavin PIN by contrasting, via accuracy versus coverage curves, the weakest links in the modules against the manually curated BGS set of high-confidence physical binary interactions that represent direct protein associations rather than indirect ones [6]. Modules, or highly interconnected regions in a network, can be considered to contain enrichments of triangles in which three nodes are completely interconnected. The IDBOS-Gavin PIN contains 43,054 triangles, 17 times more than that in a commensurate random network (averaged over 1000 realizations). The weakest link in a triangle is the interaction having the lowest CS score. As such, the weakest links in the IDBOS-Gavin PIN are good candidates for possible indirect associations. We compiled all the weakest links, and their corresponding CS scores, in the IDBOS-Gavin PIN and evaluated this interaction subset against the BGS set (Figure 4E). While this weakest-link IDBOS-Gavin set performs slightly worse than the complete IDBOS-Gavin scored data (Figure 2A), it is of very similar quality to the Y2H data sets (Figure 4E). Therefore, the weakest links in the IDBOS-Gavin PIN most likely represent direct interactions, indicating that the observed modularity is not an artifact arising from misrepresenting indirect associations as direct interactions.

**Figure 5:**
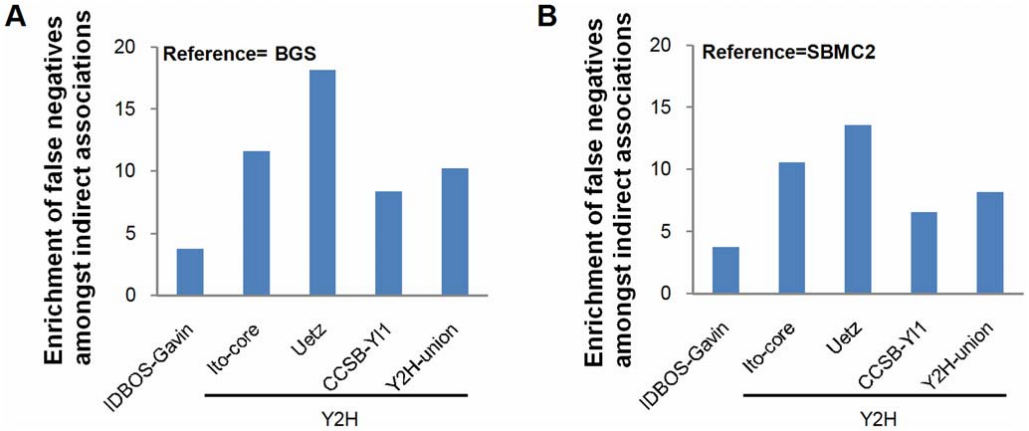
Indirect associations in the IDBOS-Gavin PIN and Y2H data sets are enriched with false negatives. An indirect association occurs when two non-interacting proteins share an interaction partner, e.g., A and B represent an indirect association in the case of A–C–B. Indirect associations form a subset of all non-interactions. A false negative is defined as a non-interaction that is curated as a direct physical interaction in a reference set: (A) BGS, (B) SBMC2 (see Materials and Methods). The fraction of indirect associations that are false negatives (actual) was compared with the fraction of all non-interactions that are false negatives (expected). Enrichments were computed as ratios of actual/expected.

We next turned our attention to the undetected interactions in the experiments. For Y2H data sets, they denote protein pairs that did not restore a transcription factor activating expression of a reporter gene, while in our analysis of AP/MS data they represent non-specific, or low-scoring, protein associations in the purifications. A false negative is here defined as an undetected interaction that is curated as a direct physical interaction in a reference set. The BGS and SBMC2 curated data sets were considered to be appropriate references (see Materials and Methods). Good candidates for false negatives are undetected associations between two proteins who share an interaction partner, i.e., indirect associations arising from cases of A–C–B, where two proteins A and B are not found to associate but both are evinced to interact with protein C. The fraction of these indirect associations that are false negatives (actual) was compared with the fraction of all undetected interactions that are false negatives (expected). Enrichments were computed as ratios of actual/expected. Enrichments for the IDBOS-Gavin and Y2H PINs were greater than three (Figures 5A–B); therefore, the results suggest that in all these data sets indirect associations are more likely to be false negatives, at least as categorized by the BGS and SMBC2 references. The enrichment is least for the IDBOS-Gavin PIN but substantial for the Y2H interaction sets with the data of Uetz et al. [5] showing the largest proportion of possible missed interaction detections.

These findings imply that the high modularity observed in the IDBOS-Gavin PIN was not a result of misrepresenting indirect associations as direct interactions and, in fact, indicate that the modularity would be enhanced if curated high-quality binary interaction data was included. The results affirm that the IDBOS scoring procedure is able to adequately distinguish between the direct and indirect associations in the purification data. We also found that the modularity in Y2H interaction data sets may be underrepresented, particularly the data of Uetz et al. [5], as the constituent indirect associations were significantly enriched with false negatives. It must be stressed that these inferences were largely based on the assumption that the BGS [6] and SBMC2 [24] data sets comprise veritable direct binary physical protein interactions. We note that the BGS data has recently been utilized to demonstrate that the qualities of high-throughput Y2H data sets are substantially better than those of high-throughput AP/MS data sets [6].

### High-Confidence AP/MS PINs Show Assortative Mixing

**Figure 6:**
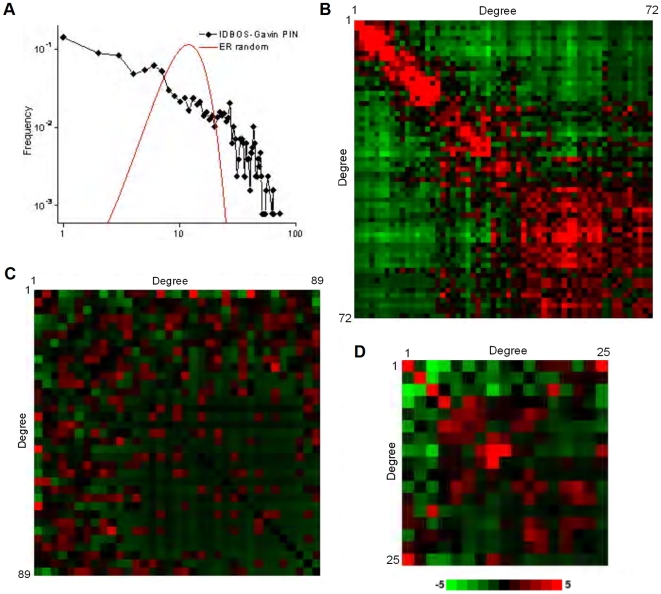
High-confidence AP/MS interaction data shows assortative mixing while Y2H interaction data shows disassortative mixing. (A) Power-law-like degree distribution of the IDBOS-Gavin PIN and for a commensurate completely random Erdös-Rényi (ER) graph. Enrichments (Z-scores) of interaction frequencies, relative to commensurate, degree-preserving random networks (10^4^ realizations) between pairs of degrees in the (B) IDBOS-Gavin PIN, (C) Y2H-union data set [6], and (D) BGS curated interaction set (see Materials and Methods). Most red indicates Z≥5 (overrepresented) and most green indicates Z≤−5 (underrepresented).

Having established that the observed modularity in the high-confidence IDBOS-Gavin PIN is likely a result of direct interactions, we probed the network features further. As noted above, the IDBOS-Gavin PIN has a power-law-like degree distribution that is substantially different from that of a completely random Erdös-Rényi graph having the same number of nodes and edges (Figure 6A). The observed non-random degree distribution is not surprising, but welcome, since it is well established that many real-world networks, including biological, have power-law-like degree distributions [26],[33],[34]. With respect to biological networks and PINs, previous studies have found that they are disassortative [21],[26], meaning that interactions tend to occur between two nodes, or proteins, that have very different degrees, i.e., hubs, or proteins having very many interactions, prefer to connect to proteins having very few interactions. A consequence of disassortativeness is that hubs avoid interacting with each other and prefer to spread out in a PIN rather than clump together centrally. We investigated the connectivity in the IDBOS-Gavin PIN by computing interaction frequencies for pairs of degrees. The significances of the frequencies, computed as Z-scores illustrated in Figure 6B, were evaluated by comparison against frequency distributions resulting from 1000 realizations of commensurate, degree-preserving random networks. The diagonal nature of the degree-degree frequency distribution is immediately apparent. High-degree proteins prefer to interact with each other while low-degree proteins avoid interacting with hubs. In fact, the IDBOS-Gavin PIN appears highly assortative - interactions tend to occur between proteins having very similar degrees. To confirm this property, we evaluated the degree-degree correlation coefficient (−1≤*r*≤1), whereby a negative value indicates disassortativeness and a positive value signifies assortativeness [26],[27]. As expected, the IDBOS-Gavin PIN has a considerably positive correlation coefficient of 0.62, confirming its inherent assortative nature. For comparison, a commensurate degree-preserving random network has an average correlation coefficient of −0.02 (SD = 0.01) for 1000 realizations; this value is slightly negative due to the exclusion of self interactions. The previous finding of disassortativity [21] was based on a study of the Y2H interaction data of Ito et al. [4]. Significances of degree-degree interaction frequencies for the Y2H-union data set [6] are shown in Figure 6C and it is clear that this network contains weak disassortative mixing (*r* = −0.08) - hubs generally prefer to interact with low-degree proteins and there is only a slight diagonal propensity. Therefore, we confirm the previous finding [21] that Y2H interaction data appears disassortative while high-quality AP/MS interaction data constitutes significant assortative mixing. These findings are in line with the observations noted above, whereby the modularity in the IDBOS-Gavin PIN is likely due to direct interactions while the modularity in Y2H data sets may be underestimated due to missed interaction detections. This inference is reflected in the significances of the degree-degree interaction frequencies in the manually-curated BGS set [6] shown in Figure 6D, where significant simultaneous disassortative and assortative elements result in an ‘X’ pattern. In fact, the degree-degree correlation coefficient for this interaction data was essentially zero (*r* = 0.004), indicating that the disassortative and assortative mixing effects are nearly identical.

## Discussion

We have developed a statistical approach to measure the affinity for two proteins to co-purify in an AP/MS data set. The method is not based on machine-learning techniques and, therefore, requires no external reference data set. As such, it is applicable to any current and future AP/MS data set regardless of how much curated information is available. Our scoring mechanism is distinct from previous approaches in that it utilizes random baselines derived by thoroughly shuffling, or exchanging, prey proteins. Therefore, the approach preserves the numbers of proteins in the individual purifications and takes into account the experimentally discerned affinities of the bait proteins.

The procedure was applied to two recent yeast AP/MS studies [8],[9] and it was shown that the derived scored interaction data sets were enriched with specific, or discriminating, protein associations as compared to random profiles. It was also demonstrated that known high-quality direct physical interactions had significantly high scores. The scored interaction data sets were further evaluated by comparisons against four diverse high-quality reference data sets and it was generally found that our scoring system performed superior to previous scoring schemes [16],[17]. Additionally, our scored interaction data sets were the only ones that almost consistently outperformed experimental Y2H interaction sets [4]–[6], including when contrasted against the curated BGS set which represents high-confidence direct physical binary associations [6].

Although it is generally accepted that AP/MS experiments detect preponderant non-specific (transient) protein interactions, our analyses reveal an underlying specificity for protein associations, i.e., a subtle preference for proteins to form functional interactions. While ours and previous studies [8],[9],[16],[17] have implied such a specificity by showing that high-scoring associations generally appear in manually curated reference sets, we have further demonstrated that the experimental score distributions are distinct from commensurate random profiles. The random profiles for three different AP/MS data sets are almost identical revealing a consistency in our scoring approach. Additionally, the experimental score distributions have enhanced tails in the high-score region, thereby demonstrating enrichments of interaction specificity in each experimental data set. The interplay between non-functional and functional interactions was recently explored using Y2H interaction data and it was conjectured that the impact of non-functional interactions upon biochemical efficiencies of specific complexes was near the tolerable limit [19]. Since our analyses of AP/MS data provide specificity profiles, we hope that our scored interaction data sets may reveal further insights into the non-functional/functional interaction dynamics occurring in the cell.

From the scored data sets we derived, using 5% false-discovery rates, corresponding high-confidence PINs. We selected that derived from the AP/MS data of Gavin et al. [8] for further network study after inferring that it contained the highest specificity. We stress that our determination of specificity in the AP/MS data sets was based on our score-enrichment and abundance analyses and did not consider other mitigating factors. Therefore, we are reluctant to make firm conclusions regarding the data sets of Gavin et al. [8] and Krogan et al. [9]. Our high-confidence PIN derived from the data of Gavin et al. [8] was shown to be highly modular and strikingly distinct to a commensurate random degree-preserving network. Additionally, we demonstrated that the high modularity was not a consequence of misinterpreting indirect associations as direct interactions. We propose that the lack of modularity in Y2H PINs is the result of enrichments of false negatives due to undetected interactions between indirectly associating proteins.

In line with these findings, the network structures of our high-quality AP/MS and Y2H PINs were found to be significantly different - our AP/MS PIN shows strong assortative mixing while Y2H PINs show weak disassortative mixing. A consequence of assortative mixing in AP/MS data sets is that high-degree proteins (hubs) prefer to interact with other high-degree proteins; however, the disassortative mixing in Y2H PINs means that hub proteins avoid each other and instead connect to low-degree proteins. As a result of the network connectivity differences, our high-quality AP/MS data set appears more modular than Y2H interaction sets. However, the curated BGS set shows both, and in equal measure, assortative and disassortative mixing, suggesting that both elements are actually present in comprehensive cellular interaction networks.

It remains to be seen whether the enriched levels of specificity observed in the yeast AP/MS data sets also exist in AP/MS data for other organisms, particularly those that do not have multiple compartments. The modular nature of the specificity discovered here for the yeast AP/MS data indicates a clear biological propensity for the formation of individually functioning complexes. Maximum insights into the nature of this selective clustering will be gained by mapping biological properties of the proteins, such as function and compartment locality, upon the scored interaction data. While we have carried out such analyses, these results will be presented and discussed at a later time. Previous studies of AP/MS data suggest that high-confidence interactions most likely occur between proteins having the same function and locality [16] and we can confirm that the modules involve proteins of similar function (results not shown). Therefore, the observed assortative mixing by degree also exists for biological function. Comprehensive analysis of the mixing patterns by function and compartment in high-quality AP/MS and Y2H PINs should yield further insights into the natures of interaction detection of both platforms.

We anticipate that our scored yeast data sets will be valuable for further biological discovery and that our technique will be useful for the analysis of current and future AP/MS data sets for a variety of species.

## Supporting Information

Table S1 IDBOS scores for pairs of proteins having co-occurrences greater than one in the AP/MS data of Gavin et al. [8].
(0.80 MB TXT)

Table S2 IDBOS scores for pairs of proteins having co-occurrences greater than one in the AP/MS (MALDI) data of Krogan et al. [9].
(0.69 MB TXT)

Table S3 IDBOS scores for pairs of proteins having co-occurrences greater than one in the AP/MS (LCMS) data of Krogan et al. [9].
(2.41 MB TXT)

Figure S1 Evaluations comparing the scoring schemes of IDBOS (this work) and Collins et al. [16] when applied to the purification data of Krogan et al. [9]. IDBOS-Krogan scores were obtained by combining the individual IDBOS-Krogan (MALDI) and IDBOS-Krogan (LCMS) scores. When an interaction occurred in both data sets, a commensurate score was obtained by multiplying P-values (conversion from Z scores). Four diverse reference interaction data sets were used (A) BGS; (B) PCA; (C) SBMC2; (D) MIPS. See Materials and Methods for full descriptions of these references. Also shown is the scored data of Hart et al. [17] (determined by multiplying individual results across the Gavin et al. [8], Krogan et al. [9], and Ho et al. [7] AP/MS data sets) and evaluations for Y2H data sets of Yu et al. [6] (CCSB-YI1), Ito et al. [4] (core subset), Uetz et al. [5], and a union of these data sets [6] (Y2H-union).
(0.78 MB PDF)

Figure S2 Depictions of interaction data sets generated from the raw AP/MS data of Gavin et al. [8] using the (A) spoke (bait-prey tabulation) and (B) matrix (bait-prey and prey-prey tabulations) models.
(0.31 MB PDF)
