# Supplementary material for: A Novel Scoring Approach for Protein Co-Purification Data Reveals High Interaction Specificity
Source: PLoS Comput Biol. 2009 Sep 25;5(9):e1000515. doi: 10.1371/journal.pcbi.1000515 (PMC2738424; doi:10.1371/journal.pcbi.1000515)
Supplement: Figure S1 — Evaluations comparing the scoring schemes of IDBOS (this work) and Collins et al. [16] when applied to the purification data of Krogan et al. [9]. IDBOS-Krogan scores were obtained by combining the individual IDBOS-Krogan (MALDI) and IDBOS-Krogan (LCMS) scores. When an interaction occurred in both data sets, a commensurate score was obtained by multiplying P-values (conversion from Z scores). Four diverse reference interaction data sets were used (A) BGS; (B) PCA; (C) SBMC2; (D) MIPS. See Materials and Methods for full descriptions of these references. Also shown is the scored data of Hart et al. [17] (determined by multiplying individual results across the Gavin et al. [8], Krogan et al. [9], and Ho et al. [7] AP/MS data sets) and evaluations for Y2H data sets of Yu et al. [6] (CCSB-YI1), Ito et al. [4] (core subset), Uetz et al. [5], and a union of these data sets [6] (Y2H-union). (0.78 MB PDF) [file pcbi.1000515.s004.pdf]

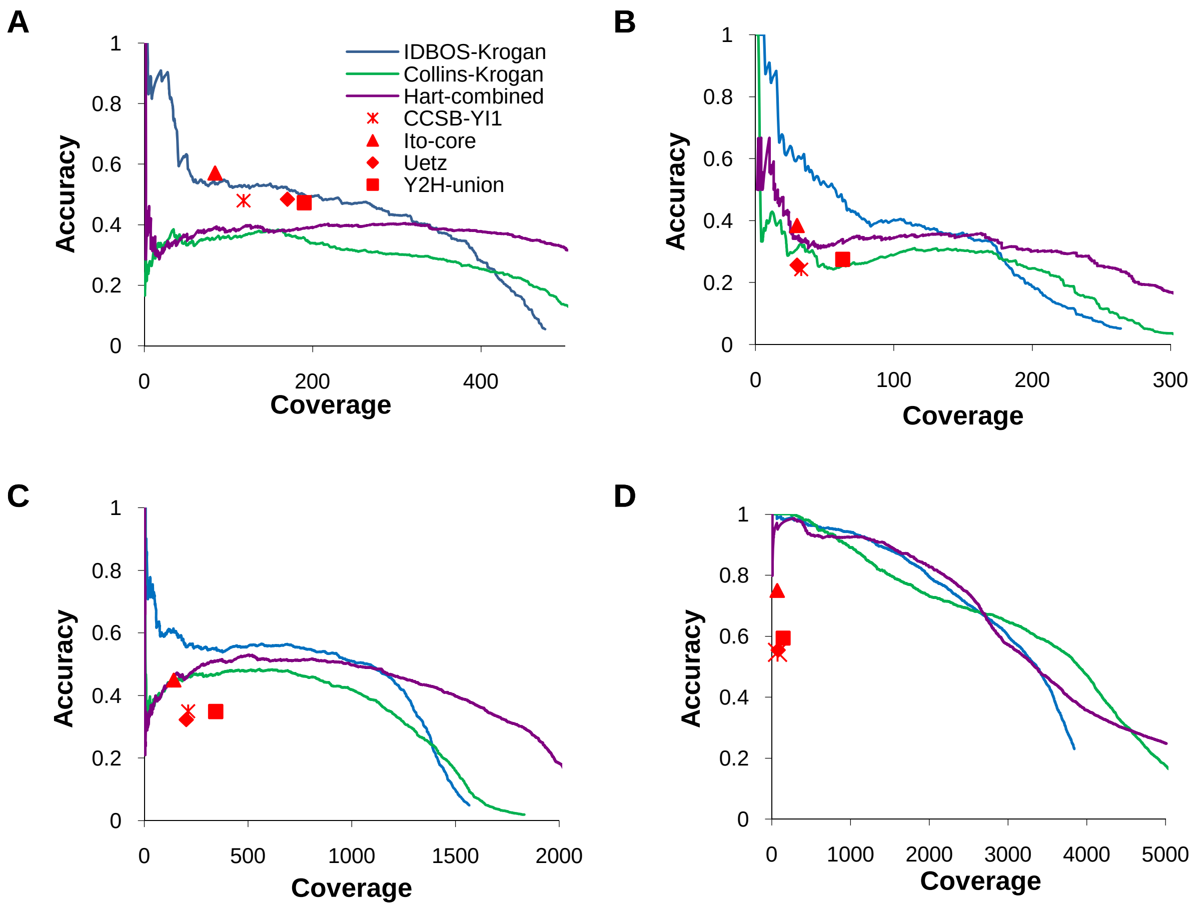

Figure S1. Evaluations comparing the scoring schemes of IDBOS (this work) and Collins et al. [16] when applied to the purification data of Krogan et al. [9]. IDBOS-Krogan scores were obtained by combining the individual IDBOS-Krogan (MALDI) and IDBOS-Krogan (LCMS) scores. When an interaction occurred in both data sets, a commensurate score was obtained by multiplying P-values (conversion from Z scores). Four diverse reference interaction data sets were used (A) BGS; (B) PCA; (C) SBMC2; (D) MIPS. See Materials and Methods for full descriptions of these references. Also shown is the scored data of Hart et al. [17] (determined by multiplying individual results across the Gavin et al. [8], Krogan et al. [9], and Ho et al. [7] AP/MS data sets) and evaluations for Y2H data sets of Yu et al. [6] (CCSB-YI1), Ito et al. [4] (core subset), Uetz et al. [5], and a union of these data sets [6] (Y2H-union).
