# Supplementary material for: A Novel Scoring Approach for Protein Co-Purification Data Reveals High Interaction Specificity
Source: PLoS Comput Biol. 2009 Sep 25;5(9):e1000515. doi: 10.1371/journal.pcbi.1000515 (PMC2738424; doi:10.1371/journal.pcbi.1000515)
Supplement: Figure S2 — Depictions of interaction data sets generated from the raw AP/MS data of Gavin et al. [8] using the (A) spoke (bait-prey tabulation) and (B) matrix (bait-prey and prey-prey tabulations) models. (0.31 MB PDF) [file pcbi.1000515.s005.pdf]

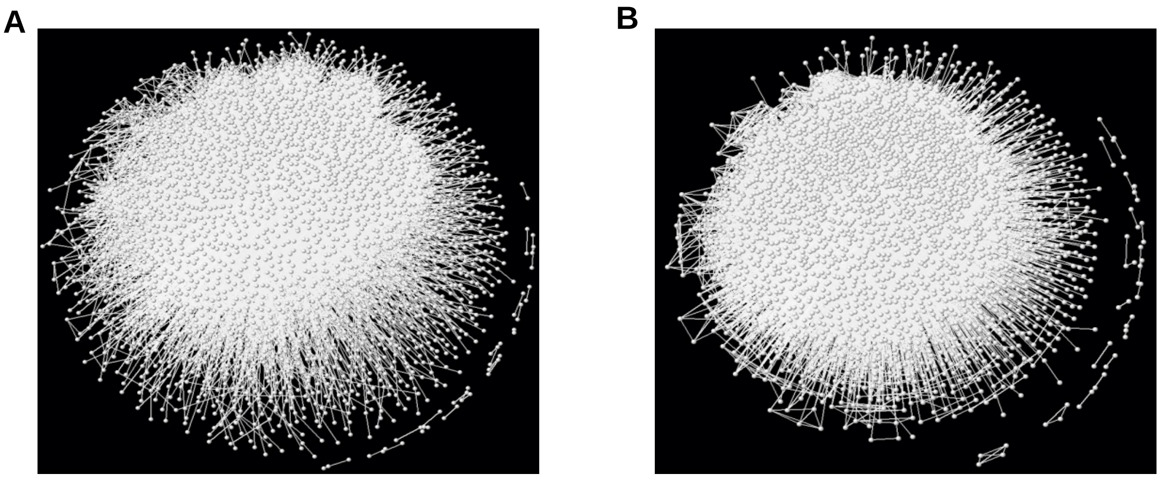

Figure S2. Depictions of interaction data sets generated from the raw AP/MS data of Gavin et al. [8] using the (A) spoke (bait-prey tabulation) and (B) matrix (bait-prey and prey-prey tabulations) models.
